# Tobacco : Its Use and End

**Published:** 1882-02

**Authors:** 


### TOBACCO—ITS USE—AND END.

Some years ago a youth, aged sixteen, while at college, had a severe tooth-ache; his grandmother gave him a piece of tobacco to put in his mouth to remove the pain; it did so, and from that time he chewed it for nine or ten years, almost incessantly. While at college, and during a three years' course at a theological seminary, he applied himself closely, paid no attention to the rules of health, took little or no exercise, and soon after he was settled as a clergyman, he became dyspeptic, and during warm weather suffered greatly from depression of spirits and mental lassitude, which seemed to incapacitate him for the proper discharge of ministerial duty; and as this duty had to be performed, he began to use brandy and water to dispel the lassitude, but only on occasions of making a public effort at first; in three or four years, he felt that the use of spirits of some kind was a daily necessity. If omitted for a single day, he could not bring his mind to bear on any subject. About this time he began to find that he could not calculate with certainty upon the effects of the stimulus as to time or amount; occasionally it almost overpowered him, and as irretrievable disgrace would have been the result, he substituted laudanum, some twenty drops, thrice a day, or often enough to keep up a uniform sensation. Whenever the stimulus was about exhausted, he would begin to gape; this was the signal for a new supply. After a while, laudanum was not strong enough, and he began to take the pure opium, the amount being increased from time to time, until he found himself taking half-an-ounce a week, which is two hundred and forty grains, or nearly thirty-five grains a day equivalent to three or four tablespoonsful of laudanum, which is thirty times more than a dose for a full-grown man. "At this time," he writes, "I became greatly disordered in body, not merely through the opium, but also through the baneful habits connected therewith. I sat at my books and papers, day after day, from breakfast until past midnight, in a hot study filled with smoke from a cigar, kept perpetually alight. I suffered martyrdom from costiveness, often going nearly a week without a passage. Sometimes, too, I got into a physical state which opium would not stimulate, and then I was compelled to employ alcohol! But alcohol, acting upon opium-drugged nerves, is exceedingly apt to produce maniacal intoxication." At this juncture he made an effort to break up these habits. For ten days and nights he was not conscious of one moment of sleep; he was half delirious for several days; the blood in his veins felt like boiling water, and rushed with such fury to the head as to make him feel as if it would split open. For a whole year he was as feeble as a child, "a walking depository of aches and distressing sensations;" he then quitted his profession and retired to the country to study law; he was attacked with neuralgia in the head and face,—this at length became unendurable, and he was advised to take morphine and quinine, which fixed the habit of using opium as firmly as ever. For two years he made no decided effort to escape from his habits, when he applied for admission into an asylum, and for eighteen months never felt well, free from pain, "for one remembered day." Troubles came, and he returned to the use of his opiate, and continued for two years, when he found himself using sixty grains of sulphate of morphine, that is, nearly nine grains a day, or thirty-six times more than a common dose for a strong man, enough to destroy life in a few hours He now took charge of a country parish, where he remained for two years, but found it impossible to perform his official duties, mentally or physically, without the aid of a quarter of an ounce of morphine, and sometimes more, a week, which is equal to some seven hundred grains of opium or sixty drops to a dram or teaspoonful, equaling ten tablespoonsful of laudanum a day, or twenty-four hundred drops; and when it is remembered that half a drop of laudanum is considered a dose for a young infant, the reader may have some idea of the magnitude of the daily portion. He is now striving to do with from half an ounce to an ounce of opium a week, averaging some five table spoons of laudanum a day. Time only can tell the end of this strife—most probably it will be the gutter and the grave.

Will any young man, especially any aspirant for the ministry after reading this statement of actual facts, dare allow *the first,* or *another* particle of tobacco, or any other mere stimulant, ever pass his lips? You are commanded to pray every day, "lead us not into temptation;" can you thus pray as often as the morning comes, that you shall not be abandoned to the power of temptation, and yet that very day, perhaps that very hour, first expose and then yield yourself to it? If so, then it well becomes you to investigate anew, "what manner of spirit ye are of."

The Editor feels that any comment on the history just given would but weaken it, and he yields the young reader to the power of fact and conscience.

---

"Diseases is very various—very. The Doctor tells me that poor old Mrs. Haze has got two buckles upon her lungs! It's dreadful to think of--'tis really. The diseases is so various! One day we hear of people's dying of 'hermitage of the lungs,' another of 'brown-creatures;' here they tell us of the 'elementary canal' being out of order, and there about the 'tear of the throat;' here we hear of the "newrology in the head,' and there of an 'embargo' in the back. On one side of us we hear of a man getting killed by getting a piece of beef in his 'sarcofagus,' and there another kills himself by 'deskevering his jocular vein.' Things change so that I don't know now to subscribe for any thing now-a-days. New names and 'rostrums' take the place of the old, and I might as well throw my old yerb bag away."

